# Single-Center Real World Experience with the VARIPULSE Platform for Pulsed Field Ablation of Atrial Fibrillation, Atrial Flutter, and Redo Procedures

**DOI:** 10.3390/jcm15010028

**Published:** 2025-12-20

**Authors:** Nizar Andria, Ziad Abuiznait, Mussa Saad, Samer Yousef, Sergey Keselman, Ibrahim Marai

**Affiliations:** 1 The Lydia and Carol Kittner, Lea and Benjamin Davidai Division of Cardiovascular Medicine and Surgery, Cardiovascular Department, Tzafon Medical Center, Poriya P.O. Box 15208, Israel; 2 The Azrieli Faculty of Medicine, Bar Ilan University, Safed P.O. Box 1589, Israel

**Keywords:** pulsed field ablation, VARIPULSE, atrial fibrillation, atrial flutter

## Abstract

**Background/Objectives**: Pulsed field ablation (PFA) is increasingly used for pulmonary vein isolation (PVI). One of the emerging single-shot PFA catheters is the variable-loop circular catheter (VARIPULSE™, Biosense Webster, Inc.) which is fully integrated into a three-dimensional mapping system. However, the evidence for the feasibility of ablation of non-pulmonary vein targets is still limited using the VARIPULSE catheter. In this study, we summarize our experience in PVI and mapping/ablation of non-pulmonary vein sites in patients with atrial fibrillation (AF) and complex atrial substrate and arrhythmias using the VARIPULSE catheter. **Methods**: All patients with paroxysmal or persistent AF who underwent catheter ablation using the VARIPULSE catheter were retrospectively included. PVI was performed in all patients. Spontaneous or inducible atrial flutters were mapped and ablated. Empiric lines were performed at the operator’s discretion. Acute outcomes and complications were analyzed. **Results**: the study included 60 patients; 25 (41.6%) were females and mean age was 67.15 ± 9.01 years. Thirty four (60%) had persistent AF and six (10%) patients had atrial flutter as the initial rhythm during the index procedure. All patients had PVI using the PFA as per protocol. Most of the patients (76.7%) had non-pulmonary vein ablation sites; posterior wall isolation was performed in 25 (41.7%) patients, roof line in 9 (15%) patients, anterior line in 16 (26.7%) patients, cavotricupsid isthmus in 11 (18.3%) patients and superior vena cava isolation in two (3.3%) patients. Overall, 27 patients had atrial flutters during the index procedure that were mapped and ablated using the VARIPULSE catheter. All had termination of atrial flutter except for one patient. Major complications were not detected. **Conclusions**: Mapping and ablation of atypical atrial flutter and non-pulmonary vein targets are feasible and safe using the VARIPULSE platform.

## 1. Introduction

Atrial fibrillation (AF) stands as the most widespread cardiac arrhythmia, contributing substantially to global health and economic burdens [1]. This condition not only affects patient morbidity and mortality but also significantly increases healthcare costs and resource utilization [1]. Given its prevalence and associated risks, effective treatment modalities are crucial for improving patient outcomes and alleviating the societal impact of AF [2]. Historically, thermal ablation techniques, such as radiofrequency (RF) and cryoablation, have been the mainstay for rhythm control in AF, yet they carry inherent risks of collateral tissue damage [3]. Pulsed field ablation (PFA) has emerged as a novel alternative, leveraging electroporation to selectively ablate myocardial tissue while minimizing thermal injury to surrounding structures [4]. This method employs ultrarapid electrical pulses to induce cell death through electroporation, representing a significant advancement over conventional thermal energy sources. This technological advancement is particularly promising for pulmonary vein isolation (PVI), where the risk of esophageal or phrenic nerve injury associated with thermal ablation modalities is a significant concern. However, despite its advantages, the long-term durability of PFA lesions and the electrophysiological findings during redo procedures after PFA-based pulmonary veins isolation remain areas requiring further investigation [4]. The integration of three-dimensional electroanatomical mapping (3D-EAM) with PFA systems may offer enhanced procedural guidance, potentially mitigating the complexities associated with left atrial anatomy and reducing fluoroscopy dependency during catheter manipulation [5]. This is particularly relevant given that the procedural times for PFA and cryoballoon ablation have been observed to be shorter compared to RF ablation, albeit sometimes at the cost of higher fluoroscopy exposure [6,7].

There are several commercially available PFA technologies including focal and multi-electrode catheters for AF ablation [8,9,10]. Several studies also showed the feasibility and efficacy of PFA for non-pulmonary vein (non-PV) targets in persistent AF and patients with atypical atrial flutter [11,12,13]. One of the emerging single-shot PFA catheters is the variable-loop circular catheter (VARIPULSE™, Biosense Webster, Inc.). The VARIPULSE catheter is fully integrated into 3D-EAM systems and features a variable-loop design [14]. The system enables creating 3D electroanatomical maps during sinus and during atrial arrhythmia, which can help to define and ablate areas of slow conduction and/or critical isthmuses for atrial re-entries. However, the evidence for feasibility and efficacy of ablation of non-PV targets is still limited using the VARIPULSE catheter.

In this study, we summarize our experience in PVI and mapping/ablation of non-PV sites in patients with AF and complex atrial substrate and arrhythmias using the VARIPULSE catheter.

## 2. Methods

### 2.1. Study Population

All patients with paroxysmal or persistent AF who underwent catheter ablation using the variable-loop circular catheter (VARIPULSE™, Biosense Webster, Inc., Irvine, CA, USA) from February 2025–September 2025 were retrospectively included.

### 2.2. Patient Selection

Patients were evaluated in different outpatient clinics and referred for catheter ablation of AF. Patients referred for a first-time ablation for paroxysmal AF underwent ablation using the modality selected at the operator’s discretion. Patients with recurrent AF, atypical atrial flutter, or persistent AF underwent ablation using the VARIPULSE™ catheter.

### 2.3. Ablation Procedure

All patients provided informed consent for the ablation procedure. Deep sedation was used in all except the first three patients, who had ablation under general anesthesia. Oral anticoagulation was continued uninterrupted.

Femoral venous access was obtained under ultrasound guidance. Intravenous (IV) heparin (50 IU/kg) was administered before, and again immediately after trans-septal puncture to achieve an activated clotting time (ACT) of 350 s. A deflectable sheath (Visigo, Biosense Webster, Inc., Irvine, CA, USA) was advanced into the left atrium, through which the VARIPULSE catheter was introduced.

In the first 20 patients, irrigation at 4 mL/min was used, including during PFA applications. In subsequent patients, irrigation at 30 mL/min was applied during PFA applications. A 3D electroanatomical map of the left atrium and pulmonary veins (PVs) was created, and the ostium of each PV was annotated.

In patients presenting with atrial flutter at baseline or induced at the end of procedure by atrial pacing or in those who developed atrial flutter following PFA delivery, electroanatomical mapping and entrainment mapping of the atrial flutter circuit was performed using the VARIPULSE catheter.

### 2.4. Ablation Strategy

For PVI, after creating 3D-EAM of the left atrium and PVs, a minimum of two ostial and two antral PFA applications were delivered per vein, with catheter rotation between applications, as recommended by the manufacturer [15]. In brief, the catheter was advanced slowly out of the sheath in the middle of the left atrium, while turning its handle to allow the loop to curve. The VARIPULSE catheter remained in the blood pool until the tissue contact indicator (the Tissue Proximity Indication on the CARTO™ System) training was complete to ensure adequate catheter–tissue proximity during PFA application [15]. Rotational mapping techniques were utilized to ensure complete circumferential lesions and to avoid gaps around the ostial (closed loop) and antral (open loop) PVs.

Posterior wall isolation was performed at the operator’s discretion in patients with persistent AF. Posterior wall isolation was performed by overlapping applications covering all the posterior wall based on the 3D-EAM. In patients with atrial flutter, additional PFA applications were delivered according to the presumed location of the critical isthmus based on activation and entrainment mapping. Left atrial roof ablation following PVI was performed by placing the VARIPULSE catheter in the left superior PV and overlapping applications were delivered sequentially along the left atrial roof toward the right superior PV, with gradual catheter repositioning via sheath retraction and rotation of PFA catheter [14]. Overlapping sequential PFA applications were delivered for ablation of anterior line (from right superior PV to mitral valve), lateral mitral isthmus line (from left inferior PV to mitral valve), and Cavotricupid isthmus line. Electrodes which were not in adequate contact with the tissue were deactivated. Superior Vena Cava isolation was performed by only ostial applications similar to ostial PV applications.

The first PFA application was delivered after achieving an ACT ≥ 300 s, with a target ACT of approximately 350 s. Before the first PFA application, IV atropine (0.5 mg) was administered to reduce vagal effects, and IV lidocaine (1 mg/kg) was given to reduce coughing during PFA applications.

### 2.5. Data Collection and Outcomes

Demographic and clinical data were collected for all patients. Procedural variables included total procedure time, fluoroscopy time, and additional non-PV applications. Acute outcomes included the rate of PV isolation, termination of atrial flutter, and achievement of bidirectional block across additional ablation lines. Complications (vascular, cardiac, pulmonary, renal, and cerebrovascular) were documented when present.

### 2.6. Ethical Considerations

The study was approved by the local institutional ethics committee and conducted in accordance with the Declaration of Helsinki. The requirement for informed consent for inclusion in this analysis was waived due to the retrospective nature of the study.

### 2.7. Statistical Analysis

Continuous variables are presented as mean ± SD or as median when appropriate. Categorical variables are presented as counts and percentages. Chi-square test was used for comparison of continuous variables and Student’s t-test was used for comparison of categorical variables. A p-value less than 0.05 was considered statistically significant. Statistical analyses were performed using SPSS statistical software, version 28 (IBM Corp., Armonk, NY, USA).

## 3. Results

### 3.1. Patients’ Characteristics

The study included 60 patients, of whom 25 (41.6%) were female, with a mean age of 67.15 ± 9.01 years. Hypertension was present in 41 (68.3%) patients, diabetes mellitus in 23 (38.3%), and 3 (5%) had a history of stroke or transient ischemic attack. Persistent AF was present in 34 (60%) patients, and 6 (10%) patients had atrial flutter as the initial rhythm during the index procedure. Left atrial enlargement of at least moderate severity was observed in 25 (71.7%) patients, and the mean CHA_2_DS_2_-VASc score was 2.4 ± 1.5 (median, 2) (Table 1).

Heart failure and left atrial enlargement of at least moderate severity were more common among persistent AF patients compared to proxysmal AF patients (Table 1).

### 3.2. Procedural Data

All patients had pulmonary veins isolation using the PFA as per protocol without need for RF touch-up. In redo patients (20%), only non-isolated veins were targeted.

Most of the patients (76.7%) had non-PV ablation sites. The non-PV sites were targeted if there was evidence for low voltages based on electroanatomical mapping or if atrial flutter was present at baseline or induced during or after ablation.

Posterior wall isoaltion was perfromed in 25 (41.7%) patients, roof line in 9 (15%) patients, anterior line in 16 (26.7%) patients, cavotricupsid isthmus in 11 (18.3%) patients and Suprior Vena Cava isolation in 2 (3.3%) patients (Table 2). Persistent AF was more common among patients who had ablation of non-PV sites (Table 3).

**Figure 1:**
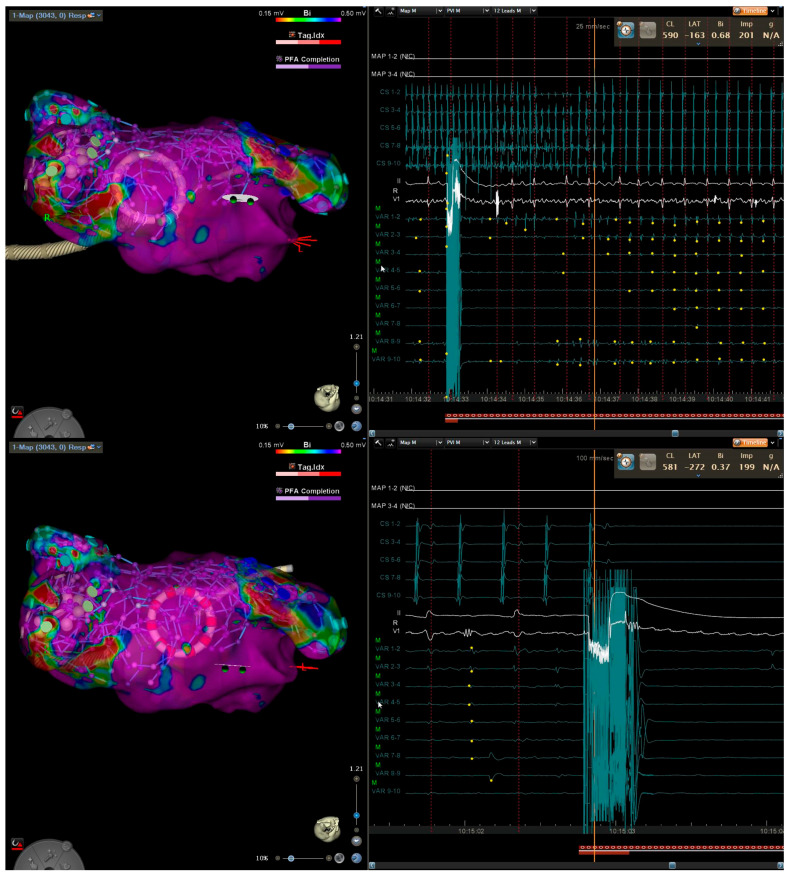
Three-dimensional electroanatomical mapping of left atrium (**left**) and EGM (**right**) in a patient with persistent atrial fibrillation lasting 12 months. After pulmonary vein isolation and posterior wall isolation using PFA, roof line connecting the suprior veins was performed with conversion first to atypical atrial flutter (**top**) and then to sinus (**bottom**).

**Figure 2:**
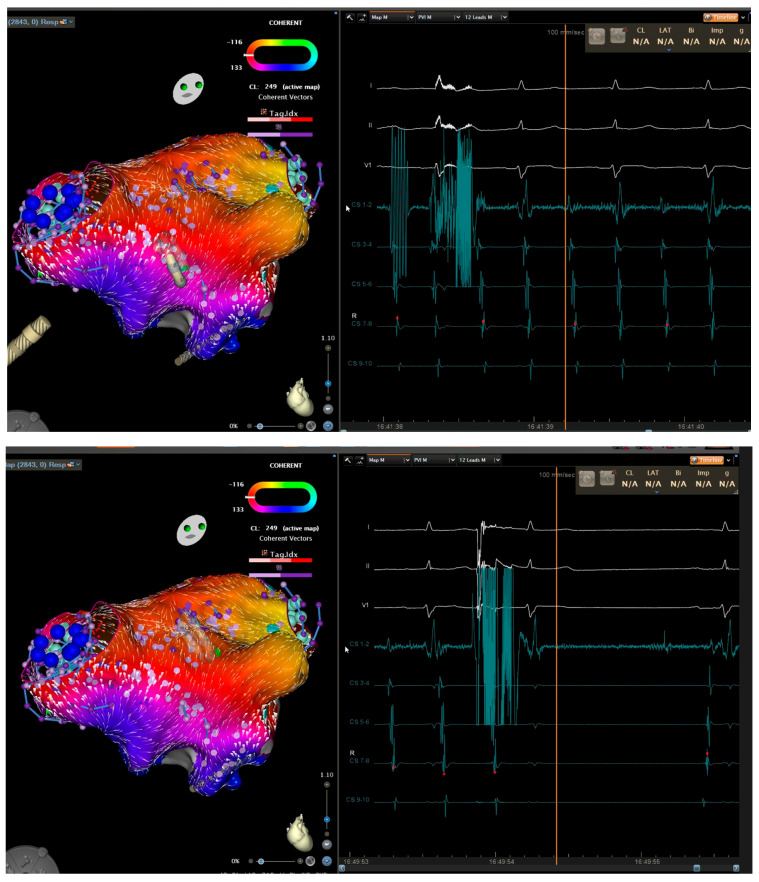
Three-dimensional electroanatomical mapping of left atrium (**left**) and EGM (**right**) in a patient with atrial flutter. Entrainment mapping and activation mapping confirmed peri-mitral atrial flutter. Anterior mitral line from right superior pulmonary vein to mitral valve caused prolongation of cycle length (**top**). Roof line connecting the superior veins terminated the atrial flutter (**bottom**).

**Figure 3:**
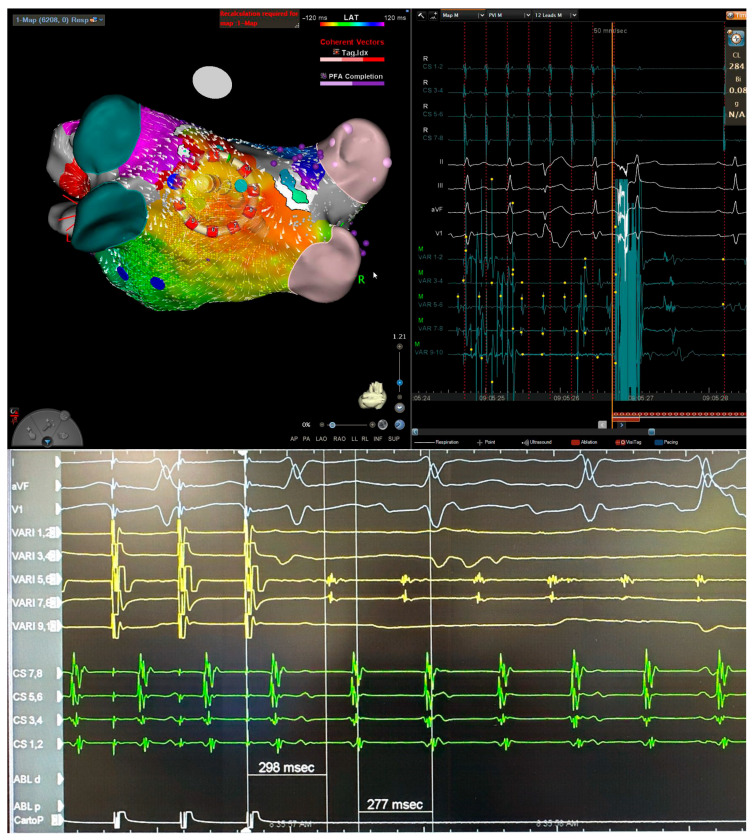
Three-dimensional electroanatomical mapping of left atrium (**left**) and EGM (**right**) (**top**) and entrainment mapping (**bottom**) using the VARIPULSE catheter in a patient with atypical atrial flutter after two previous ablations. The isthmus of flutter was located at posterior wall close to the antrum of left inferior pulmonary vein based on entrainment (**bottom**) and activation map (**top**). Application at this site terminated the atrial flutter.

Overall, 27 patients had atrial flutters during the index procedure that were mapped and ablated using the VARIPULSE catheter (Figure 1, Figure 2 and Figure 3). All atrial flutters were terminated during ablation except one atrial flutter in a patient with a history of biological mitral valve replacement who had persistent AF. In this patient, conversion to peri-mitral atypical atrial flutter was observed after isolation of PVs and posterior wall. Lateral mitral line ablation was performed with prolongation of tachycardia cycle length but without termination of the atrial flutter. Mitral isthmus block could not be achieved.

Finally, acute conduction block across all the lines was achieved in all patients except one (mitral isthmus block could be achieved). Coronary spam did not occur in any case.

### 3.3. Complications

One patient with persistent AF and reduced systolic function needed vasopressors after the end of the procedure due to sinus bradycardia and hypotension. Repeated echocardiographic tests ruled out pericardial effusion. This patient had tachycardia-induced cardiomyoypathy with moderately reduced systolic function. The procedure was performed during AF. Electrical cardioversion was performed at the end of the procedure. The profound hypotension was likely due to bradycardia and Propofol. Within several hours, the vasopressors were stopped and the patient was discharged after 24 h. One patient had a small femoral pseudo aneurysm that was treated by thrombin injection. Otherwise, no complications were reported.

## 4. Discussion

This study demonstrates the real-world feasibility, efficacy, and safety of PVI and non-PV ablation using the variable-loop circular catheter (VARIPULSE). PVI and adjunctive ablation were successfully performed in a variety of left atrial targets, including the posterior wall, roof, anterior wall, and mitral isthmus, cavotricuspid isthmus, and Superior Vena Cava. Mapping and ablation of both spontaneous and inducible atrial flutter were feasible in all patients, and no major complications occurred.

The safety and efficacy of VARIPULSE-based PVI for paroxysmal AF have been previously established in the admIRE and Inspire trials [16,17]. Additional studies have confirmed these results and proposed practical workflows for PVI using the VARIPULSE platform [15,18]. However, real-world data regarding non-PV ablation and atrial flutter mapping with VARIPULSE catheter remain limited.

The VARIPULSE catheter, a bidirectional, multielectrode device integrated into a 3D mapping system, allows for both electroanatomical mapping and pulsed-field energy delivery. Integrating PFA catheter with 3D-EAM improved visualization and assessment of catheter-tissue contact, particularly around pulmonary vein ostia, which can be challenging under fluoroscopic guidance alone [5]. These features can reduce fluoroscopy exposure and enable near-zero fluoroscopy workflows [18]. Although the system is not a high-density mapping platform and catheter maneuverability can be challenging in septal or annular regions, its variable-loop and bidirectional design make it suitable for reaching most left atrial targets.

The results of our study provides an industry-independent perspective on the real-world application of PFA technology in a high-volume center, addressing the continuous need for data on durability and reconnection patterns in a diverse patient population [19]. Our data contributes to the growing body of evidence supporting PFA as a primary modality for AF ablation while also exploring its extended utility in more challenging clinical presentations, such as typical and atypical atrial flutter, and in cases requiring repeat procedures [20].

### 4.1. Non-PV Ablation and Atypical Flutter

Yogarajah et al. [14] reported the first cases of successful cavotricuspid isthmus, Superior Vena Cava, and mitral isthmus ablation using VARIPULSE without RF touch-up, highlighting the catheter’s versatility. Dahme et al. [21] reported ablation of peri-mitral macro-reentrant atrial flutter (creating an anterior line from the left superior PV to the mitral annulus) and roof-dependent atrial flutter (connecting the superior PVs) using the VARIPULSE catheter.

In the present study, roof ablation and posterior wall isolation were successfully performed in all patients in whom they were attempted. Non-PV ablations were performed either as adjunctive therapy in persistent AF or as targeted treatment for atrial flutter. Importantly, even though mid- and long-term follow-up is not available yet, the immediate and short-term results are promising, as many of the patients in this study presented with complex arrhythmia substrates or had previous failed ablations. These findings support the feasibility and safety of VARIPULSE for mapping and ablation of both typical and atypical atrial flutter, with relatively short procedural times. Consequently, the use of VARIPULSE for atypical flutter ablation has gradually became standard practice at our center.

### 4.2. Safety and Procedural Considerations

The rate of procedural complications was very low, consistent with prior reports. One patient required vasopressor support for transient hypotension attributed to bradycardia post-conversion to sinus and Propofol administration. Deep sedation was used in most patients. Similarly, Yogarajah et al. [14] reported successful procedures without general anesthesia, and Grimaldi et al. [22] observed no procedural complications under deep sedation in the inspIRE trial cohort. Deep sedation has also proven feasible with other commercially available PFA technologies [23,24,25].

In our early experience, intravenous nitroglycerin was administered before mitral isthmus and cavotricuspid isthmus ablation to mitigate potential coronary spasm. Later, it was reserved for cases with electrocardiographic evidence of spasm, which was not observed in this cohort. After the first 20 cases, the irrigation rate was increased from 4 mL/min to 30 mL/min to prevent transient electrode temperature rise and reduce microbubble formation during PFA delivery. This approach aligns with in vitro findings demonstrating that higher irrigation rates (30 mL/min) do not meaningfully increase electrode temperature despite minor Joule heating [26]. No cerebrovascular events occurred, although vigilance remains warranted given previous reports of subclinical embolic risk.

### 4.3. Limitations

This was a single-center, retrospective analysis with no mid- or long-term follow-up. Neuroimaging was not routinely performed, as no patients developed neurological symptoms. Laboratory markers of hemolysis were not collected; however, no cases of acute kidney injury occurred. These limitations restrict the generalizability of the findings and preclude definitive conclusions regarding long-term efficacy. In addition, this study did not address the durability of non-PV lesions. In general, the long-term durability of non- PV lesions using PFA technology is not well defined, and lesion durability remains a significant limitation in all available technologies [27,28,29,30].

## 5. Conclusions

Mapping and ablation of atypical atrial flutter and non-PV targets are feasible and safe using the VARIPULSE platform. The catheter’s versatility supports its use beyond conventional PVI. Prospective studies with systematic follow-up are warranted to assess long-term outcomes and durability of ablation lesions.

## Figures and Tables

**Table 1:** Baseline characteristics.

Parameters	*n* = 60	Paroxysmal AF (*n* = 26)	Persistent AF (*n* = 34)	*p*
Female gender, *n*	25 (41.6%)	12 (46.1%)	13 (38.2%)	0.53
Age, years	67.15 ± 9.01	65.15 ± 10.85	68.67 ± 6.93	0.16
Congestive heart failure, *n*	15 (25%)	2 (7.7%)	13 (38.2%)	0.007
Hypertension, *n*	41 (68.3%)	17 (65.4%)	24 (70.6%)	0.66
Diabetes mellitus, *n*	23 (38.3%)	10 (38.5%)	13 (38.2%)	0.98
History of stroke/TIA, *n*	3 (5%)	1 (3.8%)	2 (5.9%)	0.7
Mean CHA_2_DS_2_–VA score	2.4 ± 1.5	2.1 ± 1.4	2.7 ± 1.5	0.12
Median CHA_2_DS_2_–VA score	2			
Moderate/severe left atrial enlargement, *n*	24 (40%)	5 (19.2%)	19 (55.9%)	0.004
Left ventricular ejection fraction, %	56.7 ± 9.4	56.19 ± 8.0	57.11 ± 10.4	0.7
Pacemaker, *n*	1(1.7%)	0	1	
Previous antiarrhythmic drugs, *n*	42 (70%)	17 (65.4%)	25 (73.5%)	0.49
Previous beta blockers, *n*	53 (88.3%)	24 (92.3%)	29 (85.3%)	0.4
Anticoagulation, *n*				0.7
NOAC	57 (95%)	25 (96.1%)	32 (94.1%)	
Coumadin	3 (5%)	1 (3.9%)	2 (5.9%)	

NOAC—novel oral anti-coagulant, TIA—transient ischemic attack.

**Table 2:** Procedural data.

Total Number of Isolated PVs, *n*	237 (100%)
Non-pulmonary vein ablation, *n*	46 (76.7%)
Posterior wall isolation, *n*	25 (41.7%)
Roof line, *n*	9 (15%)
Anterior line, *n*	16 (26.7%)
Lateral mitral isthmus line, *n*	5 (8.3%)
Cavotricupid isthmus line, *n*	11 (18.3%)
Superior Vena Cava, *n*	2 (3.3%)
Redo procedure, *n*	12 (20%)
Procedure time, min	91.15 ± 33.6
Fluoroscopy time, min	13.2 ± 4.9
Complications, *n*	2 (3.3%)

PV—pulmonary vein.

**Table 3:** Procedural data: PVI only versus PVI and non-PV ablation.

	PVI Only (*n* = 14)	PVI+ Non-PV Ablation (*n* = 46)	*p*
Female gender, *n*	9 (64.2%)	16 (34.78%)	0.049
Age, years	66.2 ± 7.1	67.4 ± 9.5	0.6
Persistent atrial fibrillation, *n*	2 (14.28%)	32 (69.56%)	0.0003
Procedure time, min	91.07 ± 24.88	91.17 ± 35.96	0.99
Fluoroscopy time, min	13.15 ± 2.95	13.25 ± 5.29	0.94
Complications, *n*	1 (7.14%)	1 (2.17%)	0.36

PVI—pulmonary vein isolation. PV—pulmonary vein.

## Data Availability

Data are contained within this article.
